# Proteasome Functioning in Breast Cancer: Connection with Clinical-Pathological Factors

**DOI:** 10.1371/journal.pone.0109933

**Published:** 2014-10-17

**Authors:** Elena E. Shashova, Yulia V. Lyupina, Svetlana A. Glushchenko, Elena M. Slonimskaya, Olga V. Savenkova, Alexey M. Kulikov, Nikolay G. Gornostaev, Irina V. Kondakova, Natalia P. Sharova

**Affiliations:** 1 Department of Experimental Oncology, Cancer Research Institute of Siberian Branch of Russian Academy of Medical Sciences, Tomsk, Russia; 2 Department of Biochemistry of Ontogenesis Processes, NK Koltsov Institute of Developmental Biology of Russian Academy of Sciences, Moscow, Russia; 3 Department of Pathological Anatomy and Cytology, Cancer Research Institute of Siberian Branch of Russian Academy of Medical Sciences, Tomsk, Russia; 4 Department of General Oncology, Cancer Research Institute of Siberian Branch of Russian Academy of Medical Sciences, Tomsk, Russia; 5 Department of Oncology, Siberian State Medical University, Tomsk, Russia; 6 Department of Evolutionary and Developmental Genetics, NK Koltsov Institute of Developmental Biology of Russian Academy of Sciences, Moscow, Russia; University of Alabama at Birmingham, United States of America

## Abstract

Breast cancer is one of four oncology diseases that are most widespread in the world. Moreover, breast cancer is one of leading causes of cancer-related deaths in female population within economically developed regions of the world. So far, detection of new mechanisms of breast cancer development is very important for discovery of novel areas in which therapy approaches may be elaborated. The objective of the present study is to investigate involvement of proteasomes, which cleave up to 90% of cellular proteins and regulate numerous cellular processes, in mechanisms of breast cancer development. Proteasome characteristics in 106 patient breast carcinomas and adjacent tissues, as well as relationships of detected proteasome parameters with clinical-pathological factors, were investigated. Proteasome chymotrypsin-like activity was evaluated by hydrolysis of fluorogenic peptide Suc-LLVY-AMC. The expression of proteasome subunits was studied by Western-blotting and immunohistochemistry. The wide range of chymotrypsin-like activity in tumors was detected. Activity in tumors was higher if compared to adjacent tissues in 76 from 106 patients. Multiple analysis of generalized linear models discovered that in estrogen α-receptor absence, tumor growth was connected with the enhanced expression of proteasome immune subunit LMP2 and proteasome activator PA700 in tumor (at 95% confidence interval). Besides, by this analysis we detected some phenomena in adjacent tissue, which are important for tumor growth and progression of lymph node metastasis in estrogen α-receptor absence. These phenomena are related to the enhanced expression of activator PA700 and immune subunit LMP7. Thus, breast cancer development is connected with functioning of immune proteasome forms and activator PA700 in patients without estrogen α-receptors in tumor cells. These results could indicate a field for search of new therapy approaches for this category of patients, which has the worst prognosis of health recovery.

## Introduction

Proteasomes, “ubiquitous” protease systems, regulate numerous cellular processes by protein degradation and/or peptide production in all organs and tissues including malignant tumors [1]–[5]. Among proteasome forms, the special role in tumor fate belongs to immune proteasomes. Immune proteasomes contain immune protease subunits LMP7(β5i), LMP2(β1i), and LMP10(MECL1, β2i) instead of constitutive protease subunits X(β5), Y(β1), and Z(β2) of constitutive proteasomes. Subunits X and LMP7 possess chymotrypsin-like (ChTL) activity, subunits Y and LMP2 possess caspase-like activity, and subunits Z and MECL1– trypsin-like activity [6]. However, substrate binding pockets of immune proteasomes differ from those of constitutive ones [7]. So, immune proteasomes display the altered cleavage site preference with a strong predominance to cleave behind basic or hydrophobic residues that represent the correct C terminus of a major histocompatibility complex (MHC) class I epitope. Antigenic epitopes produced from tumor proteins are transported with MHC class I molecules on the tumor cell surface for their further presentation to cytotoxic T-CD8^+^ lymphocytes. Thus, immune proteasomes display anticancer function by forming antigenic epitopes in tumor cells. However, immune proteasomes may play another role and favor cell survival by quenching the oxidative stress [8], [9].

Our previous studies revealed different patterns of immune proteasome expression in rodent tumors. In ascitic carcinoma Krebs-II having developed in mice, the level of immune subunits LMP7 and LMP2 was very low in comparison with normal tissues [10]. In carcinosarcoma Walker 256 developing in WAG rats, only the level of immune subunit LMP2 was very low [11]. These facts are connected with mechanisms allowing tumor cells to avoid immune system surveillance. On the contrary, in cells of mouse hepatocellular carcinoma, the level of immune subunits LMP7 and LMP2 considerably increased in comparison with normal cells. Perhaps, immune proteasomes assist this type of tumor cells to overpass stress conditions [12]. In this case, escape of tumor cells from immune system is connected with other mechanisms not related to immune proteasomes. Thus, each tumor type has its own pattern of immune proteasome expression depending on the functions immune proteasomes perform.

Some special proteasome functions depend on proteasome regulators, in particular, on activator PA700 (19S). This activator is responsible for binding to ubiquitinated target proteins, unfolding and directing them into the proteasome proteolytic chamber [1], [2]. Previously we discovered that PA700 expression increased in growing malignant tumors, namely, in ascitic carcinoma Krebs-II and hepatocellular carcinoma in mice [10], [12]. At the same time, PA700 expression dramatically decreased in regressing carcinosarcoma Walker 256 in Brattleboro rats with the hereditary defect of arginine-vasopressin synthesis [11]. These results show that PA700 activator is very important for protein metabolism in actively proliferating tumor cells, and its lack is related with tumor regression and disappearance.

Thus, immune proteasomes and activator PA700 are key components of rodent proteasome pool that influence tumor fate. Changes of the amount of immune subunits and activator PA700 lead to the changes of proteasome activities. On the whole, the state of the proteasome system may be a unique characteristic of the certain stage of tumor development. Thus, the apprehension of peculiarities of proteasome functioning in malignant tumors of patients may clear up the mechanisms of tumor growth and metastasis, and may be important for the development of novel approaches to anticancer therapy.

According to the data of International Agency for Research on Cancer, one of four oncology diseases that are most widespread in the world is breast cancer. Moreover, breast cancer is the one of leading causes of cancer-related deaths in female population within economically developed regions of the world [13]. So far, this disease needs detailed investigation. To investigate mechanisms of breast cancer development, it is necessary to take into consideration not only its diversity by histological tumor types and stages, but also its heterogeneity by the expression of sex steroid hormone receptors and other patient clinical-pathological features, which may influence tumor development. The additional intrigue lies in the fact that proteasomes control the functioning of estrogen α receptors (ERα). These receptors undergo ubiquitination and consequent proteasomal degradation in ligand-independent or ligand-dependent manner. Degradation of unliganded ERα is involved in the quality control of the receptor. Cyclical recruitment of E3 ligases that accomplish ubiquitination, to liganded ERα and binding of ERα to the proteasome, are necessary for transcriptional activation of estrogen-responsive promoters [14]–[17]. This fact enhances the importance of studying the relation between proteasome functioning and hormone receptor expression. The scarce published data on proteasomes in human breast cancer are mainly concerned with the comparison of the content of separate proteasome subunits and ChTL activity in tumor and control tissues [18]–[23]. The main goal of the present study was to evaluate the hypothesis whether immune proteasomes and activator PA700, which have influence on the fate of animal tumors, are connected with the development of human breast cancer. We were also interested in clarification of the question about possible relationship of total proteasome pool expression and activity with the development of human breast cancer. To answer these queries, we took into account patient clinical-pathological factors, such as expression of progesterone receptors (PR) and ERα in cancer tissue, disease stage, lymph node metastasis, histological tumor type, presence or absence of menstrual cycle, applied neoadjuvant chemotherapy, age. We discovered that disease stage was connected with the expression of activator PA700 and some forms of immune proteasomes in tumor and adjacent tissues in patients without ERα in tumor cells.

## Materials and Methods

### Ethics Statement

Breasts were surgically removed from patients with verified breast carcinomas at General Oncology Department of Cancer Research Institute of Siberian Branch of Russian Academy of Medical Sciences at the period from January 2009 up to August 2013. Informed written consent of each patient was received after full explanation of the purpose and nature of all investigation procedures.

All investigations were approved by the local ethical committees of Cancer Research Institute of Siberian Branch of Russian Academy of Medical Sciences and NK Koltsov Institute of Developmental Biology of Russian Academy of Sciences, functioning according to the 3^rd^ edition on the Guidelines on the Practice of Ethical Committees in Medical Research issued by the Royal College of Physicians of London.

### Patients and breast tissue samples

Samples of tumor and adjacent (1 cm from tumor) tissues were obtained from breasts surgically removed (106 females of 30–86 years old). For Western blot analysis and proteasome activity investigation, tissue samples were frozen at −80°C.

32 patients were treated with preoperative chemotherapy with the use of 5-Ftorouracil, Doxorubicin and Cyclophosphamide for 2–4 cycles. Accordingly to the TNM Classification of Malignant Tumors (6^th^ Edition, Union for International Cancer Control), breast carcinomas at the T_1–4_N_0–3_M_0_ stages (22/61/9/14 samples of T_1_/T_2_/T_3_/T_4_ stages and 40/37/17/12 samples of N_0_/N_1_/N_2_/N_3_ stages) were studied. Histological analysis of tumors was performed by standard method of hematoxylin and eosin staining with the subsequent use of microscope Carl Zeiss Axiostar plus 1169-151, camera AxioCam ICc 3 (Germany).

### Antibodies and reagents

Combined mouse monoclonal antibodies (mAbs) to subunits α1,2,3,5,6,7, mouse mAbs to subunit Rpt6, to immune subunit LMP2 and to immune subunit LMP7, rabbit polyclonal antibodies (pAbs) to immune subunit LMP2 and to immune subunit LMP7 were purchased from Enzo Life Sciences (UK). Mouse mAbs to β-actin, peroxidase-conjugated goat anti-mouse IgG antibodies and peroxidase-conjugated goat anti-rabbit IgG antibodies were purchased from Santa Cruz (Germany). Mouse mAbs to cytokeratin 18 were purchased from Chemicon (USA). ECL reagents were purchased from GE Healthcare (UK). Proteasome substrate N-succinyl-leu-leu-val-tyr7-amido-4-methyl coumarin (Suc-LLVY-AMC) and proteasome inhibitor Z-leucyl-leucyl-leucinal (MG132) were purchased from Sigma (USA). Peroxidase blocking reagent, LSAB2 System–HRP, mouse mAbs to ERα (clone 1D5), mouse mAbs to PR (clone PgR 636) and biotinilated anti-mouse IgG antibodies were purchased from Dako (Denmark). Hoechst 33342, Alexa Fluor 488 goat anti-rabbit IgG and Alexa Fluor 594 donkey anti-mouse IgG were purchased from Invitrogen (USA).

### Immunohistochemistry

Immunohistochemical analysis of ERα and PR expression was carried out by streptavidin-biotin method. Paraffin-embedded slices (4 µm) of primary tumors underwent dewaxing in 96% ethanol and cleansed by distilled water. Subsequently, the slices were immersed in citrate buffer (0.01 M, pH 6.0). High temperature antigen demasking was carried out in two stages: at first high-temperature microwave heating for 7 min at power 60 Watt, and 1 min cooling at room temperature, then heating for 7 min at power 40 Watt, and cooling for 20 min at room temperature. Endogenous peroxidase activity was blocked by incubating the slices in Peroxidase Blocking Reagent for 10 minutes. The slices were incubated for 1 hour at 25°C with mouse mAb to ERα or PR (1∶50). Then biotinilated anti-mouse IgG antibodies (1∶400) were placed to the slices for 10 min. The slices were washed thoroughly with PBS after each stage of the procedure. Then streptavidin-biotin complex was applied for 10 min. The antibody reaction was visualized by using a 3,3′-diaminobenzidine as a chromogen and LSAB2 System–HRP. The slices were counterstained with hematoxylin, and mounted in balsam. Microscope Carl Zeiss Axiostar plus 1169-151 (Germany) was used for image analysis. The immunoreactivity of tumor cells was evaluated from 0 to 3 according to the extent of positive staining. The intensity is in the range from 0 to 3, with 0 being no staining, 1- weak staining, 2 - intermediate staining, and 3 - intense staining. A total score (TS) of hormone receptor was defined by: TS = 3×A+2×B+1×C, where A is a per cent value of intense stained cells, B is a per cent value of intermediate stained cells, and C is a per cent value of weak stained cells. TS was ranged from 0 to 300.

For evaluation of the proteasome subunit distribution in cells, samples of tumors were fixed in 4% paraformaldehyde and frozen. Cryostat sections (10 µm) were subsequently incubated at room temperature with: 1) PBS containing 1% of BSA and 0.1% Triton X-100 for 1 h; 2) PBS containing 1% of BSA, 0.1% Triton X-100 and primary rabbit pAbs to LMP7 or to LMP2 and primary mouse mAbs to Rpt6 or to α1,2,3,5,6,7 (1∶1000) or to cytokeratin 18 (1∶100) overnight; 3) PBS containing mixture of secondary antibodies Alexa Fluor 488 goat anti-rabbit IgG (1∶700) and Alexa Fluor 594 donkey anti-mouse IgG (1∶200) for 2 h; 4) PBS containing Hoechst 33342 (1∶1000) for 20 min. After being rinsed in PBS, sections were mounted in Mowiol and were analyzed under a fluorescent microscope Leica DM RXA 2 (Germany) or confocal microscope Leica TCS SP5 STED (Germany) at the Core Facility on Cell Technologies and Optical Research Methods in Developmental Biology of NK Koltsov Institute of Developmental Biology, RAS. Images were recorded and processed with the Leica LCS software. To ensure equal illumination for all treatments, the same intensity and filter settings were used throughout. Control experiments were performed by omitting primary or secondary antibodies.

### Preparation of cleared homogenates

All procedures were performed at 4°C. Frozen samples of tumor and adjacent tissues were homogenized in a dispergator “IKA ultra turrax” (Germany) in buffer containing 50 mM Tris-HCl (pH 7.5), 100 mM NaCl, 1 mM EDTA, 1 mM dithiothreitol, 10% glycerol, 10 mM Na_2_S_2_O_5_ in the ratio 1∶6 (w:v). Homogenates were centrifuged at 10,000 *g* for 30 min. Supernatants (cleared homogenates) were used for further investigation. Protein concentration in cleared homogenates was determined by Lowry method [24].

### Determination of the proteasome ChTL activity

Proteasome ChTL activity was determined by hydrolysis of fluorogenic peptide Suc-LLVY-AMC. The reaction mixture contained 20 mM Tris-HCl (pH 7.5), 1 mM dithiothreitol, 5 mM MgCl_2_, 1 mM ATP, and 30 µM Suc-LLVY-AMC. The reaction was performed at 37°C for 20 min after the introduction of 1−5 µl of the proteasome fraction (cleared homogenate) into the reaction mixture (to the final volume of 100 µl) and stopped by the addition of 1% SDS. The resulting product was detected by a fluorimeter (Hitachi, Japan) with the excitation wavelength of 380 nm and the emission wavelength of 440 nm [25]. To calculate the contribution of extrinsic proteolytic activities, MG132 as the inhibitor of ChTL proteasome activity was used. The effect of this inhibitor was preliminarily investigated with the use of proteasomes purified from the rat liver and liberated from admixed proteases as described earlier [26]. 5 µM MG132 completely inhibited ChTL activity of purified proteasomes. So, the final calculation of ChTL proteasome activity was performed as the difference between the total and residual activities in the presence of 5 µM MG132. ChTL activity was expressed as quantity of substrate (in units of fluorimeter indication) hydrolyzed at 37°C for 20 min and normalized to 1 mg of protein.

### Western blot analysis

After SDS-PAGE in 13% gel (10 µl of cleared homogenate/100–130 µg of protein per lane), polypeptides were transferred from the gel onto PVDF membrane (Immobylon, Millipore, USA). Immunodetection was carried out in accordance with procedure for “SNAP i.d.” (Millipore, USA) with the use of primary antibodies to subunits LMP7, LMP2, Rpt6, α1,2,3,5,6,7 (1∶1500) and β-actin (1∶500).

The image analysis was performed using standard ImageJ software. The relative quantities (pixel intensity) of the immunoreactive bands on X-ray film were measured. The dependence of the pixel intensity on the amount of the protein subjected to Western blotting was evaluated preliminarily. For the further procedure, the diapason of the protein amount was chosen, in which this dependence was linear.

To compare the pixel intensity of distinct proteasome subunit in multiple samples with each other, we used some recurring samples in different gels in SDS-PAGE. Results were normalized to 1 mg of protein for comparison with ChTL proteasome activity.

### Statistical analysis

Data on each breast cancer patient were entered into appropriate database file and transferred for the analysis. The statistical analysis program *Statistica 8.0* (Statsoft, 2008) was used in the research. Initially, patient clinical-pathological characteristics: age (<45/>45 yr); menstrual cycle (+/−); histological tumor type; disease stage (T_1–4_); lymph node metastases (N_0–3_); chemotherapy (−/+), the expression of ERα and PR (−/+) were assigned in the values 1, 2, 3, …, *n*. The proteasome ChTL activity and quantities of proteasome and activator subunits in tumor and adjacent tissues from each patient were clustered into subgroups, according to values. Subgroups were ranked in the values 1, 2, 3, …, *n*. Spearman ranked order correlation analysis of all variables was performed. After that, the regression analysis based on nonlinear models was conducted. To assess regression data, we applied likelihood statistics and Wald statistics (W) (because it gives more reliable results). Simple regression analysis of generalized linear models (GLM) was also applied. To reveal the simultaneous effect of different factors on proteasome characteristics, multiple GLM analysis was performed. The multivariate research was used to examine the associations between indicated proteasome characteristics and patient clinical-pathological parameters. All predictor variables were treated as ordinal/continuous and categorical variables. Factors significant at *p*<0.05 were entered into the multivariate models. Models were examined using both no-selection and stepwise selection techniques.

## Results

### Histology of tumors and expression of sex steroid hormone receptors

Histological analysis of breast tumors revealed 88 samples of invasive ductal carcinoma (IDC), 11 samples of invasive lobular carcinoma (ILC). Besides, rare types of breast cancer, such as medullary cancer (MdC) and mucinous cancer (McC), were discovered. MdC and McC were combined in the distinct group named “other types” (7 samples).

In this study, we focused our attention on the research of α type of ER, since this type of receptors is involved in the process of cellular proliferation [27]. Breast tumors under investigation were characterized by a wide range of ERα and PR levels (TS from 0 to 285).

### Distribution of proteasome subunits in tumor tissue cells

**Figure 1:**
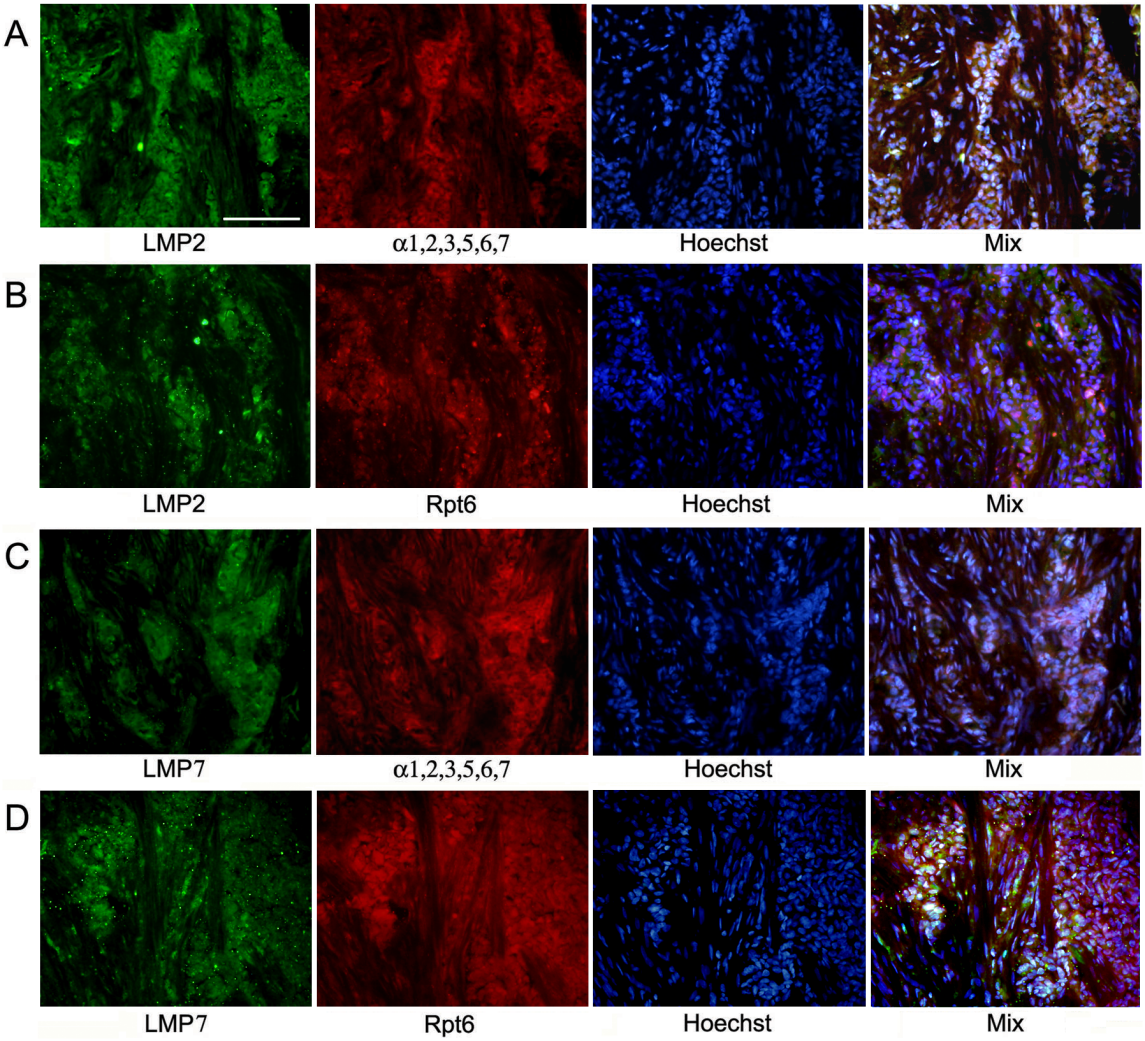
Images of cells in slices of IDC after double immunofluorescent labeling and treatment by Hoechst-33342. (Panel A) Rabbit pAbs to LMP2 subunit and mouse mAbs to α1,2,3,5,6,7 subunits, Hoechst 33342. (Panel B) Rabbit pAbs to LMP2 subunit and mouse mAbs to Rpt6 subunit, Hoechst 33342. (Panel C) Rabbit pAbs to LMP7 subunit and mouse mAbs to α1,2,3,5,6,7 subunits, Hoechst 33342. (Panel D) Rabbit pAbs to LMP7 subunit and mouse mAbs to Rpt6 subunit, Hoechst 33342. IDC, invasive ductal carcinoma. Scale bar, 100 µm.

**Figure 2:**
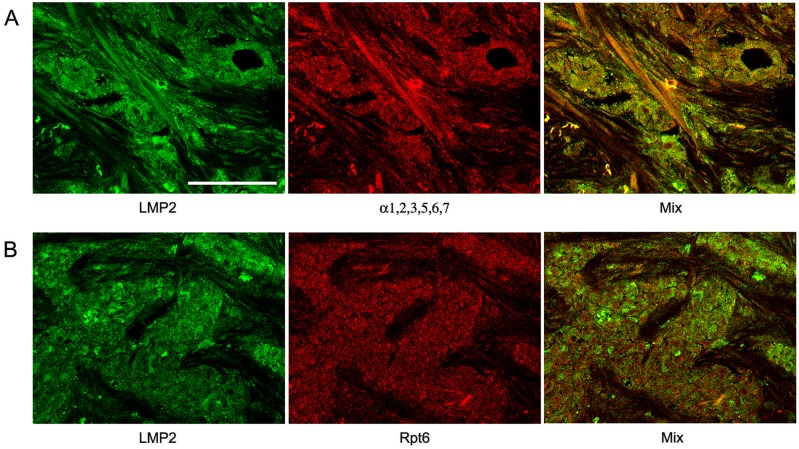
Confocal images of cells in slices of IDC after double immunofluorescent labeling. (Panel A) Rabbit pAbs to LMP2 subunit and mouse mAbs to α1,2,3,5,6,7 subunits. (Panel B) Rabbit pAbs to LMP2 subunit and mouse mAbs to Rpt6 subunit. IDC, invasive ductal carcinoma. Scale bar, 100 µm.

**Figure 3:**
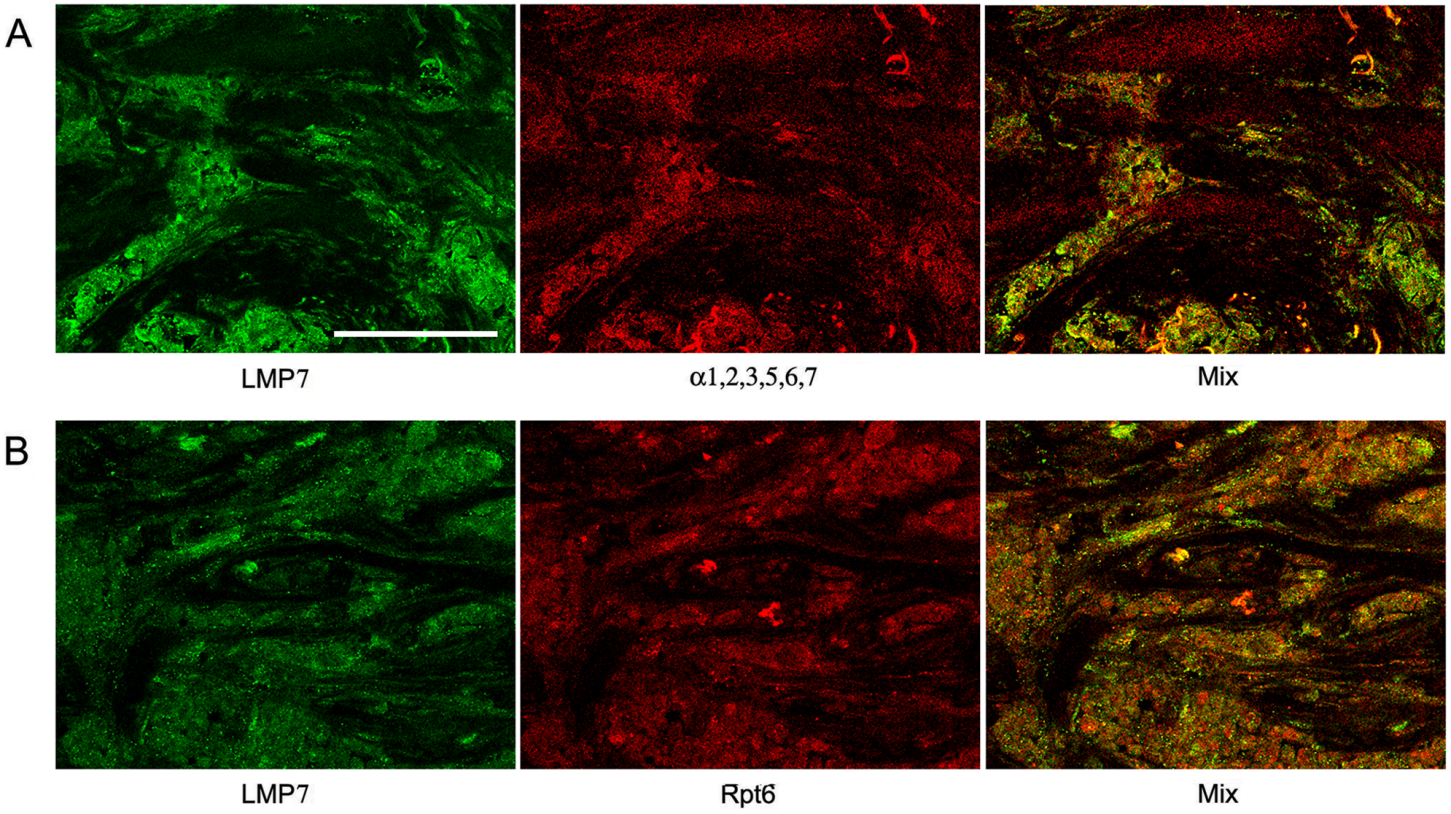
Confocal images of cells in slices of IDC after double immunofluorescent labeling. (Panel A) Rabbit pAbs to LMP7 subunit and mouse mAbs to α1,2,3,5,6,7 subunits. (Panel B) Rabbit pAbs to LMP7 subunit and mouse mAbs to Rpt6 subunit. IDC, invasive ductal carcinoma. Scale bar, 100 µm.

The distribution of the total proteasome pool was investigated according to the expression of α1,2,3,5,6,7 subunits included in all proteasome forms. The distribution of immune proteasomes containing LMP2 and/or LMP7 subunits was analyzed on the basis of the expression of these subunits. The third immune subunit MECL1 is known to be integrated into assembling proteasomes jointly with subunit LMP2 but independently from subunit LMP7 [28], [29]. So, we focused our attention on the expression of subunits LMP2 and LMP7. The distribution of proteasome activator PA700 was studied on the basis of the expression of subunit Rpt6 included in the structure of this activator. Figs. 1, 2, and 3 reflect the distribution of proteasomes and activator PA700 in IDC samples. The method of double immunofluorescent labeling discovered that cytoplasm of tumor cells contained immune proteasomes among total proteasome pool (Figs. 2A and 3A). Also, immune proteasomes were colocalized with activator PA700 in cytoplasm of tumor cells (Figs. 2B and 3B). At the same time, α1,2,3,5,6,7 subunits as well as PA700 activator were detected in nuclei of tumor cells (Figs. 2 and 3). This reflects the expression of proteasomes containing constitutive protease subunits in nuclei.

**Figure 4:**
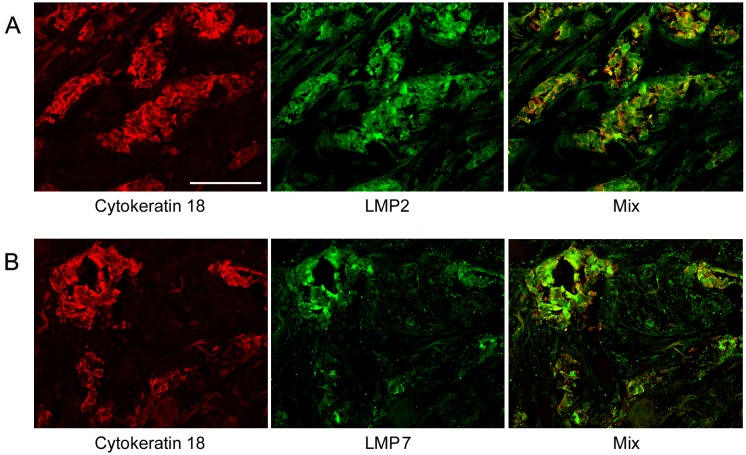
Expression of immune subunits LMP2 and LMP7 in cytokeratin 18 containing cells of IDC sample. (Panels A) Mouse mAbs to cytokeratin 18, rabbit pAbs to LMP2. (Panels B) Mouse mAbs to cytokeratin 18, rabbit pAbs to LMP7. IDC, invasive ductal carcinoma. Scale bar, 100 µm.

Note that we identified IDC cells as cellular congestions localized in ducts and fibrous stroma according to morphological characteristics detected by histological analysis. These congestions were clearly revealed by Hoechst 33342 labeling of cellular nuclei (Fig. 1). Breast tumor cells are known to express cytokeratin 18 at the high level [30]. So, we applied the additional approach to identification of tumor cells by labeling them with the use of mAb to cytokeratin 18. Fig. 4 shows that in IDC samples, cytokeratin 18 containing cells express subunits LMP2 and LMP7 to the larger extent in comparison with other cells. In Fig. 2A, one can see longitudinal and transversal slices of fibrous stroma structure besides tumor cells. Fibrous stroma cells are characterized by higher ratio of subunits α1,2,3,5,6,7 and immune subunit LMP2 in comparison with IDC cells. It means that the proteasome pool in IDC cells is enriched by immune proteasomes in comparison with fibrous stroma cells. Thus, immunohistochemical analysis showed that carcinoma samples contained mainly tumor cells enriched by immune proteasomes and PA700 activator.

### Proteasome characteristics and their connection with clinical-pathological factors

We explored ChTL activity of the total proteasome pool in all tumor and adjacent tissues. Besides, we studied the expression of subunits α1,2,3,5,6,7, LMP2, LMP7 and Rpt6 in these tissues by Western blotting and analyzed the connection of these parameters with clinical-pathological factors. Note that high levels of proteasome subunit mRNA do not always correlate with high protein levels, suggesting a specific translation mechanism which controls proteasome subunit synthesis [31]. So, our attention was paid exactly to the protein expression of proteasome subunits.

**Figure 5:**
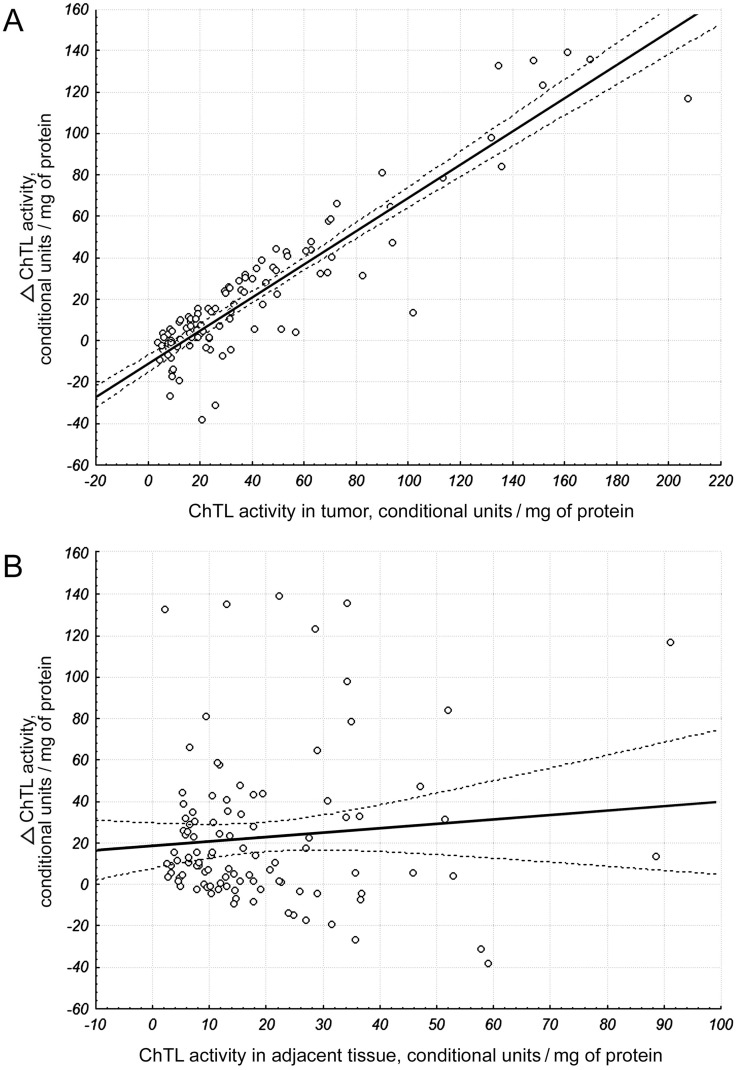
Dependence of ChTL activity difference in tumor and adjacent tissue on ChTL activity in breast samples. (A) F(1) = 519.763; *p*<0.001. (B) F(1) = 0.947; *p* = 0.333. Calculated at 95% confidence.

The wide range of proteasome ChTL activity (4–207 conditional units/mg of protein) in tumor was detected. ChTL activity in adjacent tissues lied in the range of 3–91 conditional units/mg of protein. Magnitudes of ChTL activity in the range of 113–207 conditional units/mg of protein in tumor were 2–11 times higher than in adjacent tissue. On the whole, ChTL activity was higher in tumor if compare to adjacent tissue in 76 from 106 patients. Nonparametric analysis of rank values discovered that ChTL activity in adjacent tissue was correlated with ChTL activity in tumor (ρ = 38%; *p*<0.05) and neoadjuvant therapy (ρ = 22%; *p*<0.05). Simple GLM regression analysis revealed that difference of ChTL activity in tumor and adjacent tissue depended on ChTL activity in tumor, but not in adjacent tissue (Fig. 5). For further investigation, we used maximum likelihood statistics and Wald statistics. It is shown that ChTL activity in tumor depends on index ranks of LMP7 amount in tumor (W = 3.8; *p* = 0.04). Index ranks are (1)[82−283], (2)[358−615], (3)[725−1026], (4)[1739−18568] optical density/mg of protein. However, the influence of LMP2 subunit on ChTL activity was not found (W = 0.10; *p* = 0.90).

**Figure 6:**
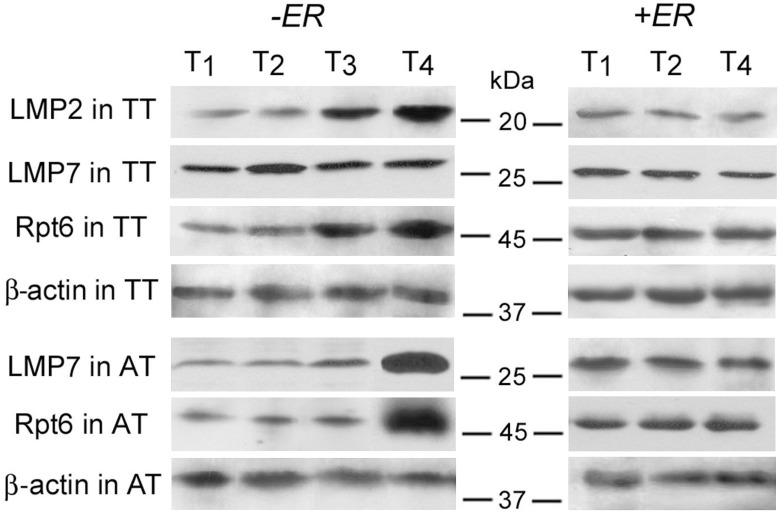
Expression of proteasome immune subunits and Rpt6 subunit in breast samples at different disease stages. Western-blotting was performed with the use of mouse mAbs to proteasome subunits LMP2, LMP7 and Rpt6; β-actin was detected as the inner control with the use of mouse mAbs to this protein. TT, tumor tissue. AT, adjacent tissue.

**Figure 7:**
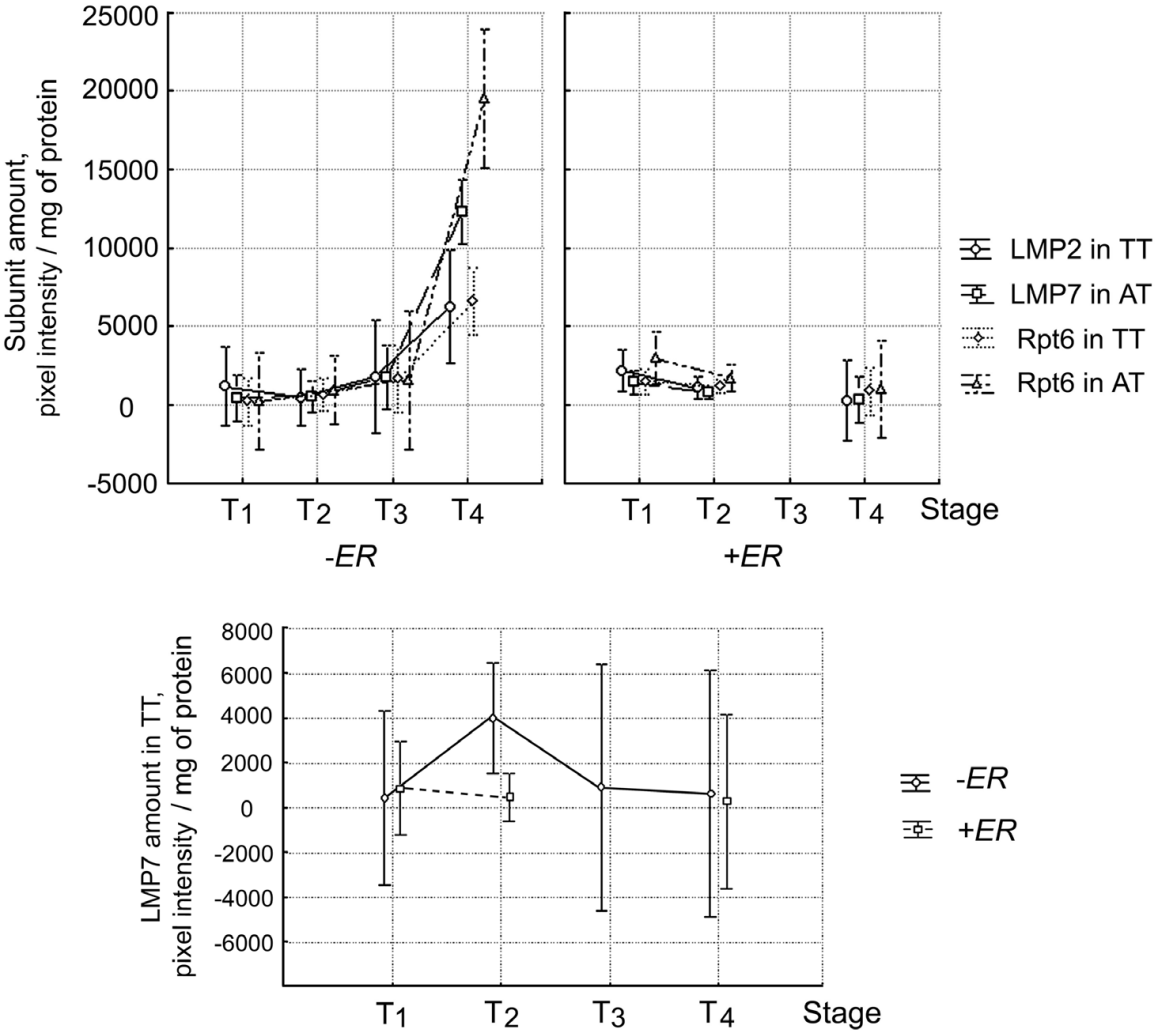
Dependence of amount of proteasome subunits in breast samples on simultaneous effect of two factors. TT, tumor tissue. AT, adjacent tissue. Factors: disease stage and ERα absence or ERα presence. Wilks lambda = 0.09; F(12; 62) = 11.911; *p*<0.001. Vertical bars denote 95% confidence interval.

Because of the heterogeneity of tumor samples by histological types and expression of sex steroid hormone receptors, it was important to evaluate the simultaneous effects of each of these factors with other clinical-pathological parameters on proteasome characteristics. The investigation was performed by multiple GLM analysis, which can reveal relationships undetectable by other statistical methods. We checked the simultaneous effect of the disease stage and factor of ERα absence or ERα presence on the quantity of subunits LMP2, LMP7, Rpt6 in primary tumor and adjacent tissue. The dependence of the quantity of LMP2 subunit in tumor, LMP7 subunit in adjacent tissue and Rpt6 subunit both in tumor and adjacent tissue on the simultaneous effect of the disease stage and factor of ERα absence or ERα presence, was revealed (Figs. 6 and 7). The statistical indicators are shown in Table 1. In ERα absence, these proteasome parameters considerably increased with tumor stage. Namely, the quantity of LMP2 subunit in tumor was higher at the stage T_4_ than at the stages T_1_ and T_2_. At the stage T_3_, it had middle value between the stages T_2_ and T_4_ but did not differ statistically. The quantities of subunits LMP7 in tumor, Rpt6 in tumor and Rpt6 in adjacent tissue were reliably higher at the stage T_4_ than at the stages T_1_, T_2_ and T_3_. On the contrary, in ERα presence all these proteasome parameters did not differ with tumor stage. We discovered similar dependence of the amount of LMP7 subunit (F = 3.15; *p* = 0.037) and Rpt6 subunit (F = 2.80; *p* = 0.05) in adjacent tissue on lymph node metastasis progression from N_2_ to N_3_, in ERα absence (data not shown). In ERα presence, these proteasome parameters did not change. Note that LMP7 subunit amount in tumor did not change reliably with tumor stage either in ERα presence or in ERα absence (Figs. 6–7). Effect of PR expression on proteasome characteristics was not revealed by any statistical method.

**Table 1:** Statistical indicators for revealed dependence of the proteasome parameters in breast samples on simultaneous effect of clinical-pathological factors.^1.^

	Simultaneous effect of clinical-pathological factors			
Expression of proteasome subunit	Disease stage and factor of ERα absence or ERα presence		Metastasis and histological tumor type	
	F (DF)^2^	*p* 3	F (DF)^2^	*p* 3
LMP2 in tumor	4.25 (2.36)	0.022	2.17 (4.29)	0.097
LMP7 in tumor	0.71 (2.36)	0.496	4.16 (4.29)	0.009
LMP7 in adjacent tissue	46.00 (2.36)	1,21×10^−10^	16.44 (4.29)	3.86×10^−7^
Rpt6 in tumor	11.30 (2.36)	1.56×10^−4^	3.77 (4.29)	0.014
Rpt6 in adjacent tissue	25.13 (2.36)	1.47×10^−7^	8.07 (4.29)	1.68×10^−4^

1 Multiple GLM (generalized linear models) analysis was applied;

2 F (DF), F-test value for indicated pair of interacting signs (Degrees of Freedom);

3 *p*, statistical significance of observed effects.

**Figure 8:**
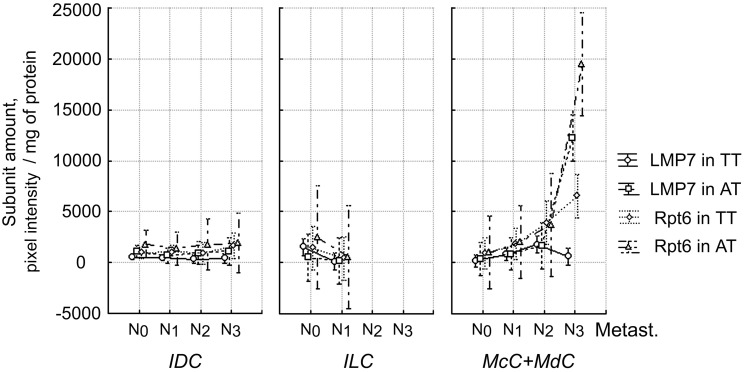
Dependence of amount of proteasome subunits in breast samples on simultaneous effect of two factors. TT, tumor tissue. AT, adjacent tissue. Factors: lymph node metastasis and histological tumor type. Wilks lambda = 0.014; F(40; 77.7) = 4.02; *p*<0.001. IDC, invasive ductal carcinoma. ILC, invasive lobular carcinoma. MdC, medullary cancer. McC mucinous cancer. Vertical bars denote 95% confidence interval.

Also, we investigated the simultaneous effect of lymph node metastasis and histological tumor type on the amount of subunits LMP2, LMP7, Rpt6 in primary tumor and adjacent tissue. The dependence of the quantity of LMP7 and Rpt6 subunits in tumor and adjacent tissue on the simultaneous effect of examined factors was revealed (Fig. 8). The statistical indicators are shown in Table 1. For ILC samples, the amount of LMP7 subunit in tumor decreased with metastasis progress from N_0_ to N_1_. For samples of “other types” (MdC+McC), the amount of LMP7 subunit in adjacent tissue and Rpt6 subunit both in tumor and adjacent tissue, increased with metastasis progress from N_0_ to N_3_.

## Discussion

The present study discovered peculiarities of proteasome functioning in breast cancer. In spite of high variability of patient clinical-pathological parameters, some important relationships were revealed. Namely, in ERα absence, the development of breast carcinoma is connected with the enhanced expression of proteasome subunit LMP2 and activator PA700 in tumor tissue. It was in tumor cells (not stroma cells) that the main amounts of subunit LMP2 and activator PA700 were fixed by method of immunohistochemistry. Relationships like these were earlier detected for thyroid cancer [32]. Perhaps, immune subunit LMP2 performs anti-stress functions in cells of these tumors and promotes their survival. The high expression of activator PA700 in dividing tumor cells is important for strict control of cell cycle and other cellular processes [33].

Besides, we detected some phenomena in adjacent tissue, which are important for tumor growth and lymph node metastasis progression in the absence of ERα in tumor. These phenomena are related to the greatly enhanced expression of activator PA700 and immune proteasomes containing LMP7 subunit. Such phenomena in adjacent tissue were also revealed in lymph node metastasis progression in the group of McC+MdC irrespectively of ERα expression. Activator PA700 and LMP7 subunit probably are involved in degradation processes to facilitate cancer expansion. This fact requires further investigation. However, at the present time, we can speculate about two probable explanations. Firstly, in cells surrounding the growing tumor, protein metabolism with proteasome participation may be reorganized and directed to maintenance of tumor growth. This consideration is founded by a known feature of breast cancer cells to subjugate fibroblasts [34]. Secondly, surrounding cells are likely to supply proteasomes to extracellular environment by exocytosis to hydrolyze matrix or other substrates and provide tumor invasion as matrix metalloproteinases do [35]. This viewpoint is based on the fact of existence of extracellular proteasome pool [36].

As the whole, the results indicate immune subunits and PA700 activator as components essential for breast cancer development. Besides, taking into account our previous data showing the connection of tumor regression in Brattleboro rats with sharply decreased PA700 level [11], we could note the significance of consideration of PA700 activator as a possible target for new drug development.

**Figure 9:**
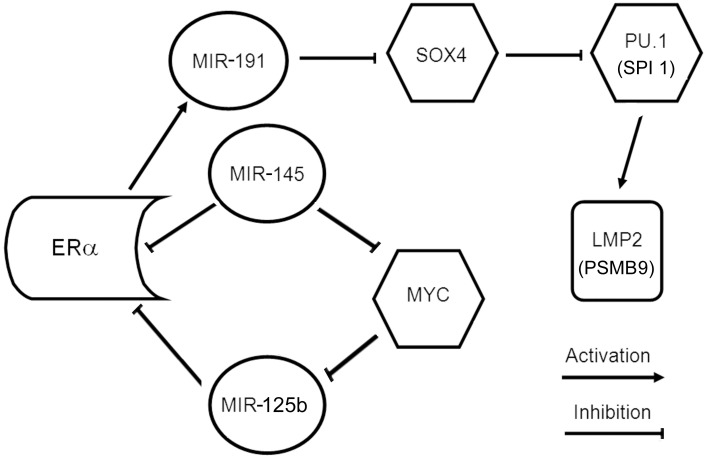
Regulatory network between ERα and proteasome immune subunit LMP2 in breast cancer. MIR, microRNA; SOX4, PU.1 (SPI 1), MYC, transcription factors. Created within open informatics site MirOB, (http://mirob.interactome.ru/pathway_navigator, 2012−2013).

The discovered complicated dependence of subunit LMP2 expression in tumor on the simultaneous effect of ERα expression and disease stage raises the question, how ERα may influence LMP2 expression in such intricate manner? It may be explained by the functioning of ERα as a factor which regulates LMP2 expression indirectly, through pathway with participation of microRNA (MIR) and some transcription factors. It is known for breast cancer that ERα induces MIR-191 expression [37]. In its turn, MIR-191 downregulates large set of genes including SOX4 in breast cancer [38]. Transcription factor SOX4 reduces mRNA levels of transcription factor PU.1 (SPI 1) in human acute myeloid leukemias [39]. This may be also the case for breast cancer, as PU.1 (SPI 1) is discovered in breast cancer too (Dataset NCBI GEO Profiles, ID: 2383767, 6783968, 77599439, 77894723, 82510260). PU.1 (SPI 1) directly binds and transactivates the promoter of PSMB9 (LMP2) [40]. Besides, MIR-145 and MIR-125b may participate in network and influence ERα expression directly [41], [42] or through transcription factor MYC [43]–[45]. Possible regulatory network between ERα and subunit LMP2 is shown in Fig. 9. Taking into consideration the fact that proteasomes control transcriptional function of ERα, we can speculate about the existence of feedback between the proteasome system and ERα in breast cancer which is regulated in the complicated network.

One of significant results is the detection of the wide range of proteasome ChTL activity in tumor samples. Tumors with magnitudes of ChTL activity in the range of 113–207 conditional units/mg of protein are likely to be most dependent on this proteasome parameter. Therefore, they may be most sensitive to inhibitors of ChTL activity. At the same time, there were plenty of patients with low ChTL activity in tumor cells at each stage of the disease. Perhaps, the influence of proteasome inhibitors on tumor cells with low ChTL activity may be commensurable with their effect on normal cells with basic low ChTL activity in different organs. Note that antitumor drug Velcade, inhibitor of proteasome ChTL activity, is applied in doses which minimize its overall-toxic action. For most solid tumor populations, including breast cancer, this drug has not shown sufficient effect [46]. Probably, one of the explanations of this fact is the presence of tumor cells with low proteasome ChTL activity.

As it was expected, ChTL activity in tumor was associated with the expression of proteasome subunit LMP7 exhibiting this activity. At the same time, the expression of LMP2 subunit was not related to ChTL activity in breast cancer, although some authors showed the connection between these parameters in mouse fibroblasts after their treatment by interferon γ [47].

Thus, the data obtained are significant for realizing the molecular mechanisms of breast cancer development. The most significant result is revealing the relation of breast cancer growth and lymph node metastasis progression with functioning of immune proteasome forms and activator PA700 in tumor and surrounding cells in patients without ERα in tumor cells. These data could indicate a field for search of new therapy approaches for this category of patients. It is important because at the present time these patients have the worst prognosis of health recovery because of futility of the therapy for them, which blocks ERα-signaling.
